# The effect of omega-3 fatty acids on central nervous system remyelination in *fat*-*1* mice

**DOI:** 10.1186/s12868-016-0312-5

**Published:** 2017-01-24

**Authors:** Elise Siegert, Friedemann Paul, Michael Rothe, Karsten H. Weylandt

**Affiliations:** 1 Division of Gastroenterology and Hepatology, Department of Medicine, Charité – Universitaetsmedizin Berlin, Campus Virchow Hospital, Augustenburger Platz 1, 13353 Berlin, Germany; 2 Experimental and Clinical Research Center, Charité – Universitaetsmedizin Berlin and Max Delbrueck Center for Molecular Medicine, Lindenberger Weg 80, 13125 Berlin, Germany; 3 NeuroCure Clinical Research Center and Clinical and Experimental Multiple Sclerosis Research Center, Department of Neurology, Charité – Universitaetsmedizin Berlin, Charitéplatz 1, 10117 Berlin, Germany; 4 Lipdomix GmbH, Robert-Rössle-Str. 10, 13125 Berlin, Germany

**Keywords:** Multiple sclerosis, n-3 PUFAs, Lipid mediators, Inflammation, Oligodendrocytes, Remyelination

## Abstract

**Background:**

There is a large body of experimental evidence suggesting that omega-3 (n-3) polyunsaturated fatty acids (PUFAs) are capable of modulating immune function. Some studies have shown that these PUFAs might have a beneficial effect in patients suffering form multiple sclerosis (MS), a chronic inflammatory demyelinating disease of the central nervous system (CNS). This could be due to increased n-3 PUFA-derived anti-inflammatory lipid mediators. In the present study we tested the effect of an endogenously increased n-3 PUFA status on cuprizone-induced CNS demyelination and remyelination in *fat*-*1* mice versus their wild-type (wt) littermates. *Fat*-*1* mice express an n-3 desaturase, which allows them to convert n-6 PUFAs into n-3 PUFAs.

**Results:**

CNS lipid profiles in *fat*-*1* mice showed a significant increase of eicosapentaenoic acid (EPA) levels but similar docosahexaenoic acid levels compared to wt littermates. This was also reflected in significantly higher levels of monohydroxy EPA metabolites such as 18-hydroxyeicosapentaenoic acid (18-HEPE) in *fat*-*1* brain tissue. Feeding *fat*-*1* mice and wt littermates 0.2% cuprizone for 5 weeks caused a similar degree of CNS demyelination in both groups; remyelination was increased in the *fat*-*1* group after a recovery period of 2 weeks. However, at p = 0.07 this difference missed statistical significance.

**Conclusions:**

These results indicate that n-3 PUFAs might have a role in promotion of remyelination after toxic injury to CNS oligodendrocytes. This might occur either via modulation of the immune system or via a direct effect on oligodendrocytes or neurons through EPA-derived lipid metabolites such as 18-HEPE.

**Electronic supplementary material:**

The online version of this article (doi:10.1186/s12868-016-0312-5) contains supplementary material, which is available to authorized users.

## Background

Multiple Sclerosis (MS) is a chronic inflammatory demyelinating disease of the CNS. In the Western world it is the main cause of disability in young adults [1] surpassed only by trauma, yet its aetiology remains elusive. Recent research has acknowledged that even at its onset MS is a neurodegenerative disease with irreversible damage to axons and neurons [2, 3]. Although the precise mechanism responsible for neuronal loss is not known, the two main risk factors for injurious processes are inflammation and demyelination.

However, recent evidence suggests that the inflammatory response to demyelination is not just a risk factor for neuronal loss, but also a necessary condition for remyelination [4–6]. It has been shown that inflammation enhances remyelination via direct effects on oligodendrocyte precursor cells (OPCs) survival, migration and differentiation [7, 8]. Also, the presence of myelin debris in CNS tissue inhibits remyelination by OPCs [9, 10]. Hence the presence of activated macrophages that clear this debris is essential for remyelination. Furthermore, activated macrophages are also capable of inducing OPCs [11, 12]. This Janus-face of inflammation has resulted in the distinction between destructive and protective inflammation, where protective inflammation is associated with enhanced remyelination and reduced neuronal loss.

Despite promising initial results, all recently developed drugs that tried to address these new findings have been unable to stop disease progression or to exert neuroprotection or even neuroregeneration [13]. Instead, some of the newly developed drugs had adverse side effects [14, 15] or unexpected drug interactions [16]. There are some suggestions that diets which are high in n-3 PUFAs and low in saturated fatty acids might stabilize disease in MS patients [17, 18]. The rationale behind this approach is that n-3 PUFAs via their lipid mediators that are also known as resolvins and protectins [19], might modulate the destructive autoinflammatory response. However, empirical evidence supporting this advice is limited. In a Cochrane review of randomized trials of dietary interventions for MS, Farinotti et al. [20] could not find a significant effect of neither omega-6 (n-6) nor n-3 PUFAs on disease progression nor relapse rate. By contrast, a recent epidemiological study from Australia suggested that the risk for central nervous system demyelination is reduced in people with a higher intake of omega-3 PUFA [21].

There have been many studies in rodents looking at the anti-inflammatory effect of PUFAs in experimental autoimmune encephalomyelitis (EAE) as an animal model for MS [22–24]. However, in all these studies the focus was on n-6 PUFAs only. So far only one study [25] has used the cuprizone model for the study of the n-3 PUFA effect in neural demyelination and remyelination and found that a diet high in salmon fish oil protected from demyelination and increased remyelination [25].

We set out to confirm the results of this first n-3 PUFA cuprizone study in the *fat*-*1* mouse model. This mouse, by expressing *fat*-*1* n-3 PUFA desaturase from C. elegans, can endogenously synthesize n-3 PUFA from n-6 PUFA without using dietary supplementation [26] thus eliminating confounding factors of diet [27]. These mice were recently used in several models of neurological disease, such as Alzheimer’s disease [28], Parkinson’s disease [29], epilepsy [30] and stroke-related brain injury [31]. In all models, when compared to wt littermates, they were protected from neuronal damage. Using the *fat*-*1* model, the cuprizone model of demyelination is superior to the EAE model, because in EAE the priming and proliferation of autoreactive T-cells might already be impeded by the differences in n-3 PUFA tissue status.

## Methods

### Animals

Transgenic *fat*-*1* mice were engineered as previously described [26]. They were subsequently backcrossed onto a C57BL/6 background at least four times. Generations of female heterozygous *fat*-*1* mice and male wt mice were then mated to obtain wt and transgenic mice from the same offspring. In this study, all transgenic *fat*-*1* mice used were heterozygous. Animals were kept under specific pathogen-free conditions in standard cages and were maintained in an air-conditioned atmosphere with a controlled 12 h light–dark cycle. They were fed a special semi-purified diet (AIN-76A containing 10% corn oil) high in n-6 and low in n-3 fatty acids. Sterile drinking water was given ad libitum. Each cage housed four to six weight-matched female mice. Mouse studies were approved by the Massachusetts General Hospital Subcommittee on Research Animal Care (Additional file 1: Figure S1).

**Fig. 1:**
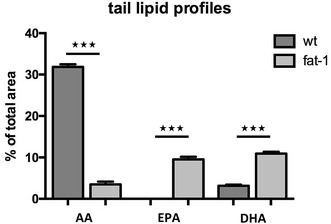
Lipid profile in wt and *fat*-*1* mouse-tails. Fatty acid composition of the tail was analysed using GC. The long-chain PUFAs AA, EPA and DHA were included in this comparison. The respective mean values and standard errors for wt animals are AA 31.9% (±0.6), EPA 0% (±0) and DHA 3.1% (±0.3). For fat-1 animals they are AA 3.5% (±0.7), EPA 9.5% (±0.6) and DHA 10.9% (±0.4). There were statistically significant differences between all three PUFAs in mouse-tails

Mice were divided into a *fat*-*1* group and a wt group according to their phenotype. For phenotyping the tip of mouse-tails (100 mg) was cut off from six-week-old mice and subjected to gas chromatographic analysis. The ratio between n-6 PUFAs [arachidonic acid (AA) and gamma-linolenic acid (GLA n-6)] and n-3 [alpha-linolenic acid (ALA), eicosapentaenoic acid (EPA), docosapentaenoic acid (DPA n-3) and docosahexaenoic acid (DHA)] was determined for each individual mouse to distinguish between wt and transgenic phenotypes. Mice that had an n-6/n-3 ratio ≤5 were assigned to the *fat*-*1* group and those with an n-6/n-3 ratio >10 were assigned to the wt group. The analysis of the three most important PUFAs, AA, EPA and DHA, are presented in Fig. 1.

### Chemically induced demyelination with cuprizone

Six experimental groups were set up to investigate the impact of endogenously altered n-3/n-6 PUFA status on remyelination following cuprizone-induced demyelination in mice (see Additional file 2: Figure S2 for details). Starting at nine weeks of age control animals (four *fat*-*1* and four wt mice) were maintained on a normal pulverized diet for five weeks, while cuprizone animals received a pulverized diet containing 0.2% cuprizone (w/w) (Bis(cyclohexanone)oxaldihydrazone, Sigma-Aldrich, St. Louis, MO, USA), also for five weeks. Animals in the cuprizone demyelination group (seven *fat*-*1* and five wt mice) were sacrificed at the end of these five weeks on the cuprizone diet and their tissue was used to assess whether there were any differences in demyelination between wt and *fat*-*1* animals. Animals in the cuprizone remyelination group (seven *fat*-*1* and five wt mice) were first fed the cuprizone diet for five weeks and were then allowed to recover for another two weeks on a normal diet. They were then sacrificed in order to see whether there were any differences in remyelination between wt and *fat*-*1* animals. Animal weight was recorded throughout the entire experiment.

All animals were sacrificed by anaesthesia with isoflurane (IsoFlo^®^, Abbott Laboratories, Abbott Park, Illinois, USA). Following their sacrifice, animals were transcardially perfused with ice-cold PBS. Brains and spinal cords were then excised and the brain was cut into three parts: the rostral ^2^/_3_ of the brain were prepared for histology; the occipital ^1^/_3_ and the cerebellum, as well as the spinal cord, were fresh-frozen and stored at −20 °C for gas chromatography (GC) analysis.

### Histology

The rostral 2/3 of the brains were first fixated in 4% PFA (Arcos Organics, Fairlawn, NJ, USA) at 4 °C for 24 h and subsequently quenched in a sucrose gradient (Sigma-Aldrich, St. Louis, MO, USA) over two days before they were fresh-frozen in Shandon M-1 Embedding Matrix^®^ (Thermo Fisher Scientific, Waltham, MA, USA) for cryosectioning. Coronal brain sections were cut at 10 µm thickness corresponding to slice 205 of the Harvard High Resolution Mouse Brain Atlas [32]. Sections were then air-dried and stored at −20 °C until further processing. Luxol Fast Blue staining was used to quantify myelin.

For Luxol Fast Blue (LFB) staining, sections were first hydrated in aqua bidest. Next, sections were rinsed in 70% ethanol and placed in 0.1% LFB (Solvent Blue 38^®^, Sigma-Aldrich, St. Louis, MO, USA) staining solution for 6 h at 60 °C. Following the 6 h of staining, sections were then rinsed in 95% ethanol, followed by aqua bidest. In the next step sections were postfixed in 1% lithium carbonate solution (Sigma-Aldrich, St. Louis, MO, USA) and subsequently rinsed in 70% ethanol and aqua bidest. This step was repeated until there was a clear differentiation between white and grey matter staining. Sections were then counterstained in 0.1% Cresyl Violet acetate solution (Sigma-Aldrich, St. Louis, MO, USA). Eventually, sections were rinsed in aqua bidest followed by 95 and 100% ethanol, then xylol and then they were coverslipped.

Stainings were evaluated with a LSM 5 Pascal confocal microscope (Carl Zeiss AG, Oberkochen, Germany). Of each brain a photo was taken of the corpus callosum at slice 205 of the mouse brain atlas. These photos were then analysed using ImageJ software by three blinded independent assessments. First, equal volumes (300,000 square pixels) of corpus callosum were selected. Within this volume the area that stained positive for myelin was then selected and the size of the myelinated area was recorded (Additional file 2: S2).

### Gas chromatography

For PUFA analysis, brain or tail tissues frozen in liquid nitrogen were ground into powder in liquid nitrogen. The powder was subjected to extraction of total lipids and fatty acid methylation by heating them at 100 °C for 1 h in a solution containing 2.5 ml hexane and 2.5 ml 14% boron trifluoride in methanol. Once the samples had cooled down to room temperature they were vortexed. Next, 1 ml water was added to the solution. In order to ensure that the concentration between the aqueous and the lipophilic phase was in equilibrium the samples were shaken by hand for 4 min. The phases were then separated by centrifugation and the lipophilic hexane phase containing fatty acid methyl esters was removed and dried under nitrogen. The fatty acid methyl ester residues were redissolved in 50 µl hexane and transferred into an autosampler vial. They were then analysed by GC using a fully automated Hewlett Packard 5890 system equipped with a flame ionization detector. Peaks of resolved fatty acids were identified by comparison with a fatty acid standard and the area under those resolved peaks represented their relative concentrations. The size of the areas was measured using a Perkin Elmer M1 integrator.

### Liquid chromatography mass spectrometry/mass spectrometry (LC MS/MS)

For analysis of monohydroxy lipid metabolites thirty milligrams ground and frozen brain tissue from wt (n = 7) and *fat*-*1* (n = 4) mice was mixed with methanol and internal standard (LTB4-d4) and hydrolysed with 300 μl of 10 M sodium hydroxide for 30 min at 60 °C. The solution was neutralized with 60% acetic acid and pH was adjusted to 6.0 with sodium acetate buffer. A solid phase extraction was performed with an anion exchange column (Bond Elute Certify II, Agilent, Santa Clara, CA) as described previously [33]. For elution, an n-hexane:ethyl acetate extraction mixture 25:75 with 1% acetic acid was used. The eluate was evaporated on a heating block at 40 °C under a stream of nitrogen to obtain a solid residue. Residues were then dissolved in 70 μl acetonitrile. An Agilent 1200 high performance liquid chromatography (HPLC) system and a solvent system consisting of acetonitrile/0.1% formic acid in water was used. The gradient elution was started with 15% acetonitrile, this was increased within 10 min up to 90% and held for 10 min. The HPLC system was coupled with an Agilent 6410 Triplequad mass spectrometer with electrospray ionization source. Analysis of lipid mediators was performed using Multiple Reaction Monitoring in negative mode and converted into an electrical signal by an electron multiplier dynode. The resulting signals were identified and quantified with the help of previously analysed standards, all from Cayman Chemical (Ann Arbor, MI, USA). Results were then analysed using Agilent Technology’s MassHunter Workstation Software Quantitative Analysis B.03.01 and Quantitative Analysis B.02.00.

Monohydroxy lipid metabolites analysed were those either enzymatically or non-enzymatically derived from the long-chain PUFAs AA, EPA and DHA. In particular, we focused on AA-derived 5-, 8-, 9-, 12- and 15-hydroxyeicosatetraenoic acid (HETE). Concerning n-3 PUFA-derived monohydroxy lipid mediators we focused on EPA-derived 5-, 8-, 9-, 12-, 15- and 18-hydroxyeicosapentaenoic acid (HEPE) and on DHA-derived 4-, 7-, 8-, 10-, 11-, 13-, 14-, 16-, 17- and 20-hydroxydocosahexaenoic acid (HDHA).

### Statistical analysis

Statistical analysis of the results was performed in Graph Pad Prism 6 (GraphPad Software, Inc., La Jolla, USA) calculating the mean values of group sizes ± standard error of the mean (SEM) and using the Mann–Whitney U test. Statistical significance was set at a level of p < 0.05. Whenever asterisks are used to describe levels of statistical significance, * stands for 0.05 > p > 0.01, ** for 0.01 ≥ p ≥ 0.001 and *** for p < 0.001.

## Results

**Fig. 2:**
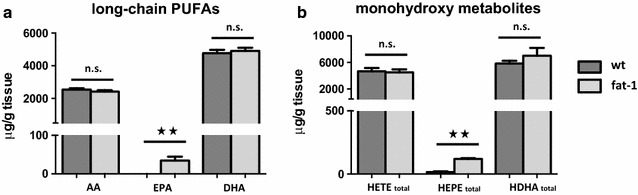
Long-chain PUFAs and their metabolites in brain tissue of wt and *fat*-*1* mice. **a** Fatty acid composition in brain tissue of wt and *fat*-*1* mice were analysed using GC. The only significant difference in n-3 PUFAs in wt and *fat*-*1* brain tissue was observed for EPA (EPA in wt cerebella 0 ± 0; EPA in *fat*-*1* cerebella 34.6 ± 19.5; p = 0.003), which was detectable only in the *fat*-*1* mice tested. AA and DHA levels in wt and fat-1 mice were wt: 2549 ± 203, 4769 ± 539; fat-1: 2418 ± 169, 4904 ± 398 respectively. **b** Lipid metabolite profiles in brain tissue of wt and *fat*-*1* mice were analysed using LC MS/MS. Similarly, levels of EPA-derived total HEPEs were significantly higher in *fat*-*1* animals. Levels of AA-derived HETEs and DHA-derived HDHAs levels were similar among wt and *fat*-*1* animals

GC was used to analyse the lipid profile of brain tissue from wt and *fat*-*1* mice. While lipid profiles of mouse-tails showed significant differences for the long-chain omega-3 PUFA AA, EPA and DHA (Fig. 1), these differences were significant only for EPA in brain tissue, which was detectable only in the *fat*-*1* mice tested (Fig. 2a). This was also reflected in the amounts of monohydroxy lipid metabolites derived from AA, EPA and DHA: While amounts of AA-derived HETEs and DHA-derived HDHAs were not significantly different, levels of EPA-derived HEPEs were significantly higher (Fig. 2b).

**Fig. 3:**
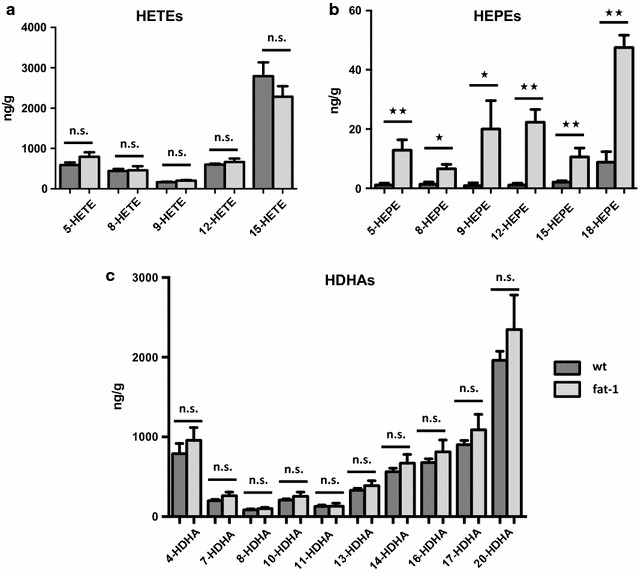
Comparison of lipid metabolite profiles in brain tissue of wt and *fat*-*1* mice. **a** Levels of AA-derived HETEs were analysed using LC MS/MS. They were relatively similar among wt and *fat*-*1* animals (presented as mean values and SEM). **b** The only significant difference in lipid metabolites between wt and *fat*-*1* brain tissue was observed for EPA-derived monohydroxy metabolites. Levels of EPA-derived HEPEs were significantly higher in *fat*-*1* animals with particularly high amounts of anti-inflammatory 18-HEPE. **c** Concerning DHA-derived HDHAs, levels were also relatively similar in the brain tissue of wt and *fat*-*1* mice with no significant differences among any of the DHA-derived metabolites

More specifically, there were no significant differences for 8-/9-HETE, as well as 5-HETE, 12-HETE and 15-HETE, which are derived from 5-, 12-, or 15-lipoxygenase action (Fig. 3a). Similarly, differences for the corresponding DHA metabolites 4-/7-HDHA, 14-HDHA and 17-HDHA were not significantly different between wt and *fat*-*1* animals (Fig. 3c). In contrast, while present only in much smaller amounts, the corresponding EPA derived metabolites were all significantly increased in *fat*-*1* mice: Most notably high amounts of 18-HEPE were found (Fig. 3b).

Given that 18-HEPE is a pathway marker of anti-inflammatory and pro-resolution mediators such as resolvin E1 and E2 [19], and an anti-inflammatory compound itself [34], we hypothesized that the difference between omega-3 PUFA in wt and *fat*-*1* mice—while only clearly discernible for EPA and its hydroxy metabolites—might lead to an anti-inflammatory and/or pro-resolution phenotype in the context of cerebral inflammation.

In the next step we thus evaluated the effect of cuprizone feeding in wt and *fat*-*1* mice. Feeding mice cuprizone had a suppressive effect on their metabolism, leading to weight loss in wt and *fat*-*1* mice (−11 and −13%, respectively). There was no significant difference between those two groups indicating that expression of the *fat*-*1* gene and the changed n-3 PUFA tissue content in *fat*-*1* mice did not interfere with the uptake and toxic metabolic effect of cuprizone in this study (Additional file 3: S3).

**Fig. 4:**
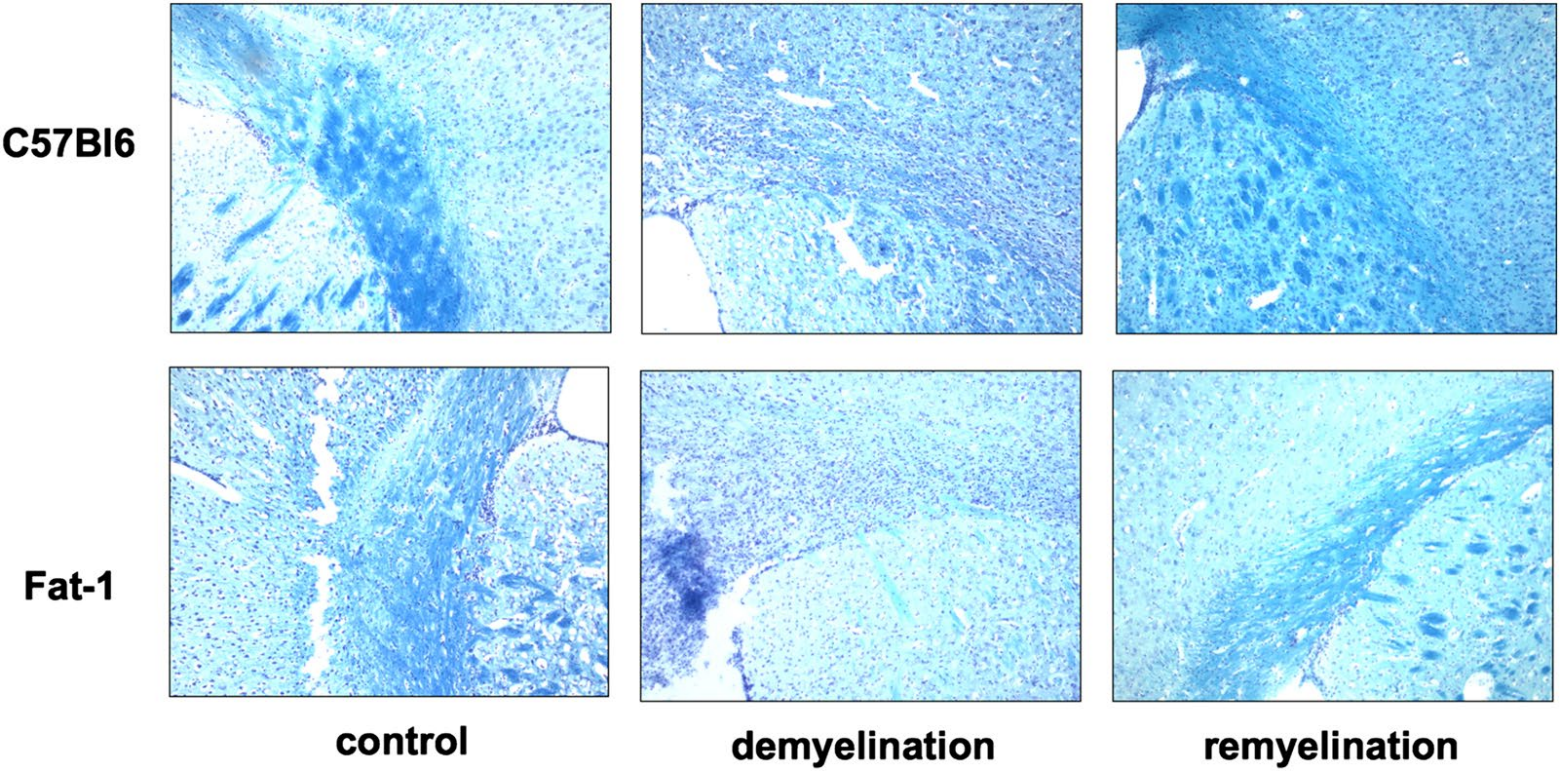
Histological Luxol Fast Blue staining for myelin was performed on cryosections of mice’s corpus callosum on slices corresponding to slice 205 of the Harvard High Resolution Mouse Brain Atlas. Pictures were taken at a magnification of ×10

The cuprizone feeding model allows to study demyelination as well as remyelination by analysing representative brain slides either directly after cuprizone feeding or later in the recovery period. Mice were sacrificed at two time points to evaluate the effect of different n-3 PUFA status on demyelination and remyelination and Luxol Fast Blue (LFB) staining was used to quantify myelin (Fig. 4). The mean volumes that stained positive for myelin were quantified as described in “Methods” (see also Additional file 3: S3).

**Fig. 5:**
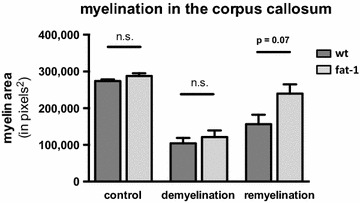
Degree of myelination in the corpus callosum. At a level corresponding to slice 205 of the Harvard High Resolution Mouse Brain Atlas, an area of 300,000 square pixels of corpus callosum tissue was selected. Within this area, the area that stained positive for myelin was selected and quantified in square pixels. From these, mean values (±SEM) were calculated for each treatment group

Next, the mean volume and the SEM were calculated for each treatment group (Fig. 5). For the control groups, there were no significant differences between wt animals and *fat*-*1* animals (wt control: mean 274,257 pixels^2^ (±4078), *fat*-*1* control: mean 287,886 pixels^2^ (±7219), p = 0.23). This was true also for the demyelination group where wt animals had a mean volume of myelin in their corpus callosum that was similar to the values observed in *fat*-*1* animals (wt demyelination 104,459 pixels^2^ (±14,398), *fat*-*1* demyelination 121,534 pixels^2^ (±18,059, p = 0.51). At the same time the cuprizone-treated groups had both significantly lower myelin staining values than the control groups (p < 0.001). In the remyelination phase there was a close miss of statistical significance between remyelinated wt animals with a lower mean volume in wt animals (156,414 ± 25,717) versus higher values in *fat*-*1* animals (239,981 ± 24,764) (p = 0.07).

## Discussion

Comparing the lipid profile of tails and brain between healthy wt and *fat*-*1* mice, we found only small differences in the FA profiles between wt and *fat*-*1* mouse brain tissue (Fig. 2a). A possible explanation for this difference is that in the CNS DHA might play such a crucial role, so that it is enriched here even in the context of low n-3 PUFA supplementation. This has been shown previously by Jeffrey et al. [35] for DHA in retinal cells. Since then it has been suggested that a similar principle might regulate trafficking of certain n-3 PUFAs to the brain [36]. In rats that are depleted of dietary n-3 PUFAs there is an increase in DHA synthesis from ALA in the liver [37, 38]. Hence one possible scenario would be that in DHA-depleted dietary states there is preferential trafficking of liver-derived DHA to the brain, amongst others through lipoprotein receptors [37].

While the total difference in DHA was small, there was, however, a significant difference of the amounts of EPA present, leading to increased amounts of potentially anti-inflammatory and anti-fibrotic 18-HEPE [39] in *fat*-*1* brains. At the same time, levels of anti-inflammatory DHA metabolites, while much higher than the EPA metabolites, were unchanged between wt and *fat*-*1* groups (Fig. 3).

In the cuprizone feeding model of demyelination damage in the brain there was no difference in the amount of demyelination between *fat*-*1* and the wt animals, possibly reflecting the fact that additional n-3 PUFA synthesis in *fat*-*1* mice was not able to confer an additional protective effect on top of the already high n-3 PUFA levels in wt brains. However, we saw improved remyelination in *fat*-*1* mice; but this difference missed statistical significance at p = 0.07. Given our relatively small sample size with an average group size of 5 animals statistical significance might be reached by increasing case numbers. Thus the data presented here suggest that an increased n-3 PUFA tissue status might lead to a mild beneficial effect of n-3 PUFAs on in vivo CNS remyelination in mice.

Our data are only partially consistent with previous data on the effect of n-3 PUFA in the cuprizone model of demyelination. Torkildsen et al. [25] had tested the effect of diets with different compositions of PUFAs on de- and remyelination in C57/BL6 mice with cuprizone-induced demyelination. They found that mice receiving a salmon-based diet suffered less demyelination in response to cuprizone than those on a cod liver or soybean oil diet and saw no differences in the degree of remyelination. The three different diets that were tested in Torkildsen’s study differed in the lipid source of their 4% lipid content. One diet contained its lipids from salmon fillets with an unknown n-3 PUFA content, the other from cod liver oil (with approx. 24 g n-3 PUFAs/100 ml cod liver oil, of which 12 g DHA and 8 g EPA) and the third from soybean oil (with approx. 50 g linoleic acid/100 ml soybean oil). Mice were started on one of these diets at four weeks of age, i.e. 4 weeks prior to cuprizone feeding. However, this study lacked direct PUFA measurements in brain tissue.

An important point of our study is that we quantified actual differences in PUFA tissue status both in the tail and in the CNS. Interestingly, despite small differences in the FA profile of wt and *fat*-*1* animal brains, we were able to see differences in the degree of remyelination between these groups. One possibility that would explain this finding is that despite similar AA and DHA profiles in the brain, there might be differences in the EPA-derived lipid mediators that are produced in the course of cuprizone-induced de- and remyelination. In this study we only established baseline measurements of hydroxyl metabolites, in which there was no difference for the DHA-derived metabolites but significant differences for the EPA-derived 18-HEPE.

Our finding of increased levels of EPA-derived 18-HEPE in the brain of *fat*-*1* versus wt mice could be interpreted in the light of other recent studies of 18-HEPE. Endo et al. [40] showed that in *fat*-*1* mice there is a selective enrichment of EPA in *fat*-*1* transgenic bone marrow cells and EPA-metabolite 18-HEPE in *fat*-*1* transgenic macrophages. Bone marrow transplantation experiments revealed that an 18-HEPE-rich environment through transplantation of *fat*-*1* transgenic bone marrow prevented macrophage-mediated cardiac remodelling via cardiac fibroblasts. This anti-fibrotic effect of 18-HEPE on cardiac fibroblasts was reproduced in vivo and in vitro. Similarly, in a study on murine macrophages 18-HEPE was able to significantly decrease macrophages’ TNF-alpha formation in vitro [33].

While there might be a tight regulation of DHA levels in the CNS, differences in EPA levels might thus lead to significant changes in anti-inflammatory lipid metabolites in the brain. Therefore, a possible explanation for the protective effect of an n-3 PUFA-enriched tissue status in CNS demyelinating disease could be a difference particularly in EPA-derived metabolites such as 18-HEPE. Based on the data from Torkildsen et al. [25], these could affect mainly macrophage function, although further experiments might be necessary to assess also the role of astrocytes in this model. Future studies will now have to assess the activity and function of macrophages/microglia found at the sites of CNS injury in the cuprizone model and to understand the effect of EPA on activity of these cells.

As compared to our analysis in mice, a meta-analysis by Farinotti et al. [20] has concluded that n-3 PUFAs did not have an effect on 292 patients with relapsing remitting MS. However, there are several arguments why a positive effect of n-3 PUFAs on remyelination in patients suffering from MS might not have shown up in the two studies that were evaluated by Farinotti and colleagues [13, 41]: Patients in both studies received EPA at 1.71 and 1.98 g/d and DHA at 1.14 and 1.32 g/d, (which is well above the general dietary recommendations by most nutritional guidelines). Both studies found that there was a rise in EPA serum level in patients receiving fish oil compared to olive oil. While Weinstock-Guttman et al. [41] in their 1-year study found that EPA levels at 6 and 9 months correlated with disability at 12 months, Bates et al. [13] found a non-significant trend between higher EPA and DHA levels due to fish oil supplements and lower disability in MS patients following two years of dietary intervention. Only Bates et al. [13] checked for effective uptake of PUFAs into adipose tissue. While there was a significant increase in EPA and DHA in adipose tissue as early as 6 months after supplementation, most of the change was only seen at 2 years on therapeutic intervention. Hence, it would be essential in any interventional study to check for effective uptake of n-3 PUFAs into relevant tissue and to choose an appropriate time frame accordingly. The data from Bates et al. [13] suggests that the time frame that was chosen in those studies might have been too short. Also, it is necessary to define what the relevant tissue is, i.e. whether differences in lipid profiles in the CNS, in adipose tissue or in immune cells confer those potential benefits. Next, a recent epidemiological study from Australia with 267 cases and 517 controls showed a reduced risk of CNS demyelination with higher intake of omega-3 PUFA [21]. Furthermore, there is the possibility that isolated n-3 PUFA supplementation might not exert the same benefit as n-3 PUFAs derived from natural nutrition. The data from Torkildsen et al. suggests that salmon-derived n-3 PUFA might be superior to cod-liver oil [24]. Given these limitations, and as concluded by Farinotti et al. [20], future studies will be necessary to settle the question of an n-3 PUFA effect in MS. This underscores that the topic of n-3 PUFA supplementation in MS warrants further investigation.

Given that our observation of increased remyelination due to increased n-3 PUFA tissue content narrowly missed significance, future mouse studies are also warranted to further establish this effect in a larger cohort of mice in the cuprizone model, as well as in other experimental MS models such as EAE.

## Conclusion

In the present study using *fat*-*1* mice endogenously forming n-3 PUFA we found a trend towards increased CNS remyelination after cuprizone damage in the context of higher EPA levels in the brain. The differences in remyelination—in the face of CNS lipid profiles differing only in EPA levels and not in DHA levels—might be due to EPA-derived metabolites such as 18-HEPE, for which anti-inflammatory and anti-fibrotic effects have been described. While DHA is more abundant in the CNS, this finding puts an emphasis on the possible role of EPA and its metabolites in CNS remyelination.

## Additional files

**Additional file 1: Figure S1.** Experimental groups. In order to assess the impact of n-3 versus n-6 PUFAs on de- and remyelination, animals were divided into six experimental groups. Ten wt and twelve fat-1 animals were put on a 0.2 % cuprizone diet for five weeks, while four wt and four fat-1 animals were fed a normal diet instead. At the end of the five weeks both control groups and the five wt and six fat-1 animals that had been fed cuprizone were sacrificed. The remaining animals were allowed to recover for another two weeks on a normal diet before they, too, were sacrificed.

**Additional file 2: Figure S2.** Quantitative analysis of histological stainings. Representative areas of the corpus callosum were marked and measured using ImageJ software. The image was cropped such that the remaining corpus callosum measured 300,000 square pixels. Within the cropped image the myelinated area was then marked and measured using ImageJ again. Values were transferred into Excel for statistical analysis.

**Additional file 3: Figure S3.** Changes of animal weight in the course of the cuprizone treatment. There are clear differences in the development of animal weight between those animals that had received the cuprizone diet and those that had stayed on a normal one. However, no significant difference could be found between wt and fat-1 animals that belong to the same treatment group.

**Additional file 4.** Data generated or analysed during this study are included in Additional file 1.
