# Supplementary figures and images for: The effect of omega-3 fatty acids on central nervous system remyelination in fat-1 mice
Source: BMC Neurosci. 2017 Jan 24;18:19. doi: 10.1186/s12868-016-0312-5 (PMC5259863; doi:10.1186/s12868-016-0312-5)

## Slide 1
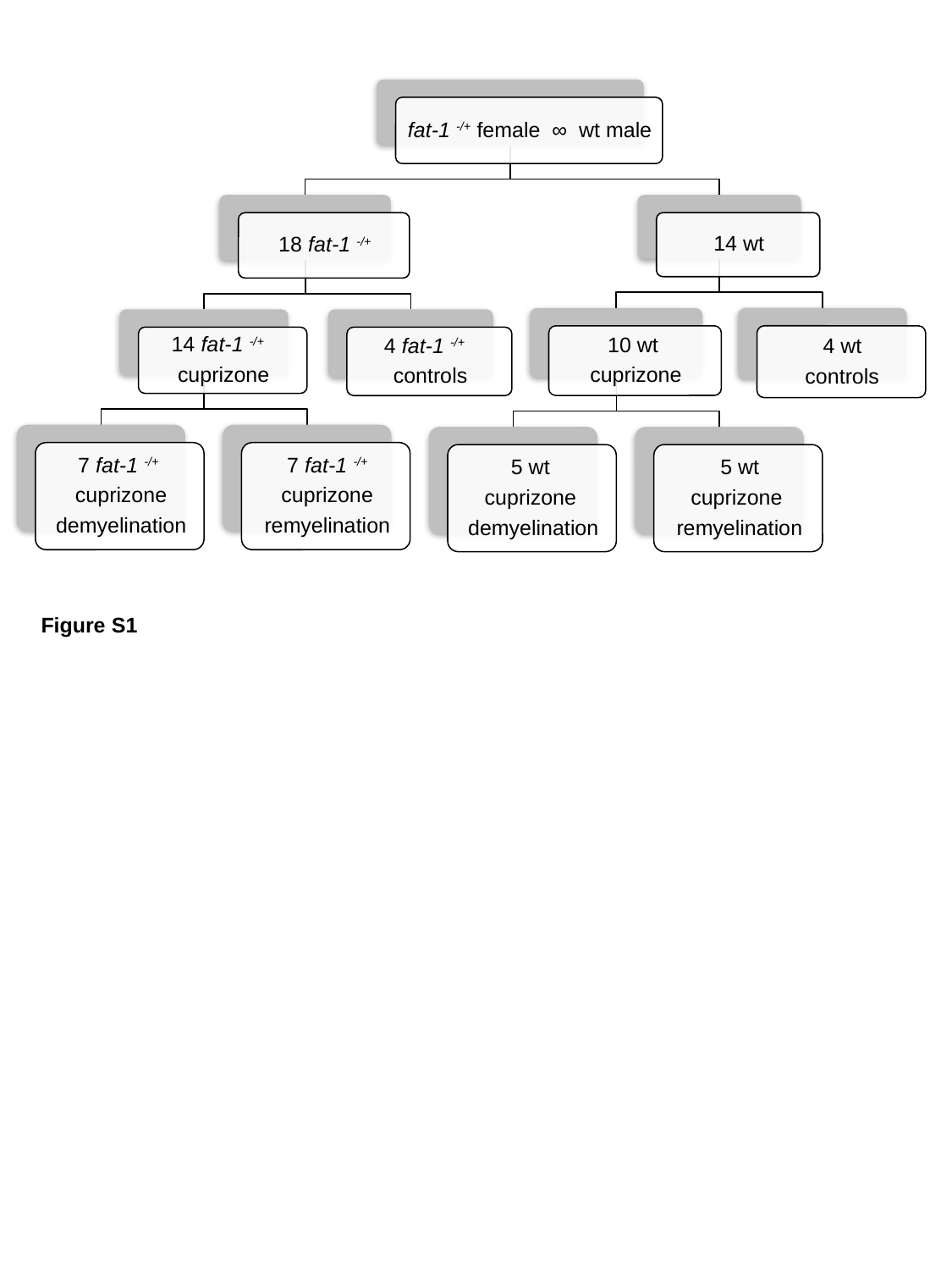

Figure S1

Supplement: Supplementary file 1 — Additional file 1: Figure S1. Experimental groups. In order to assess the impact of n-3 versus n-6 PUFAs on de- and remyelination, animals were divided into six experimental groups. Ten wt and twelve fat-1 animals were put on a 0.2 % cuprizone diet for five weeks, while four wt and four fat-1 animals were fed a normal diet instead. At the end of the five weeks both control groups and the five wt and six fat-1 animals that had been fed cuprizone were sacrificed. The remaining animals were allowed to recover for another two weeks on a normal diet before they, too, were sacrificed. [file 12868_2016_312_MOESM1_ESM.pptx]

## Slide 1
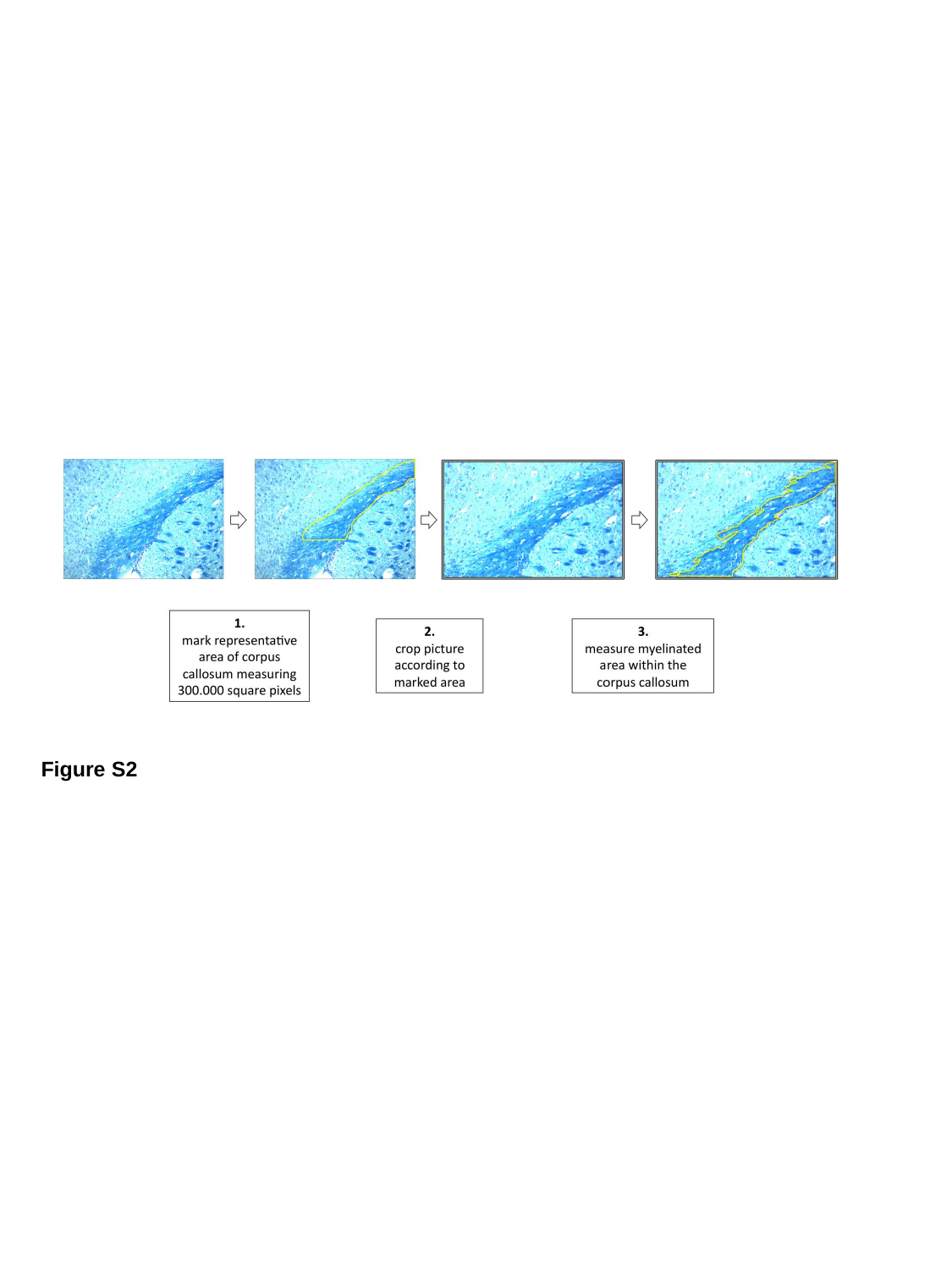

Figure S2

Supplement: Supplementary file 2 — Additional file 2: Figure S2. Quantitative analysis of histological stainings. Representative areas of the corpus callosum were marked and measured using ImageJ software. The image was cropped such that the remaining corpus callosum measured 300,000 square pixels. Within the cropped image the myelinated area was then marked and measured using ImageJ again. Values were transferred into Excel for statistical analysis. [file 12868_2016_312_MOESM2_ESM.pptx]

## Slide 1
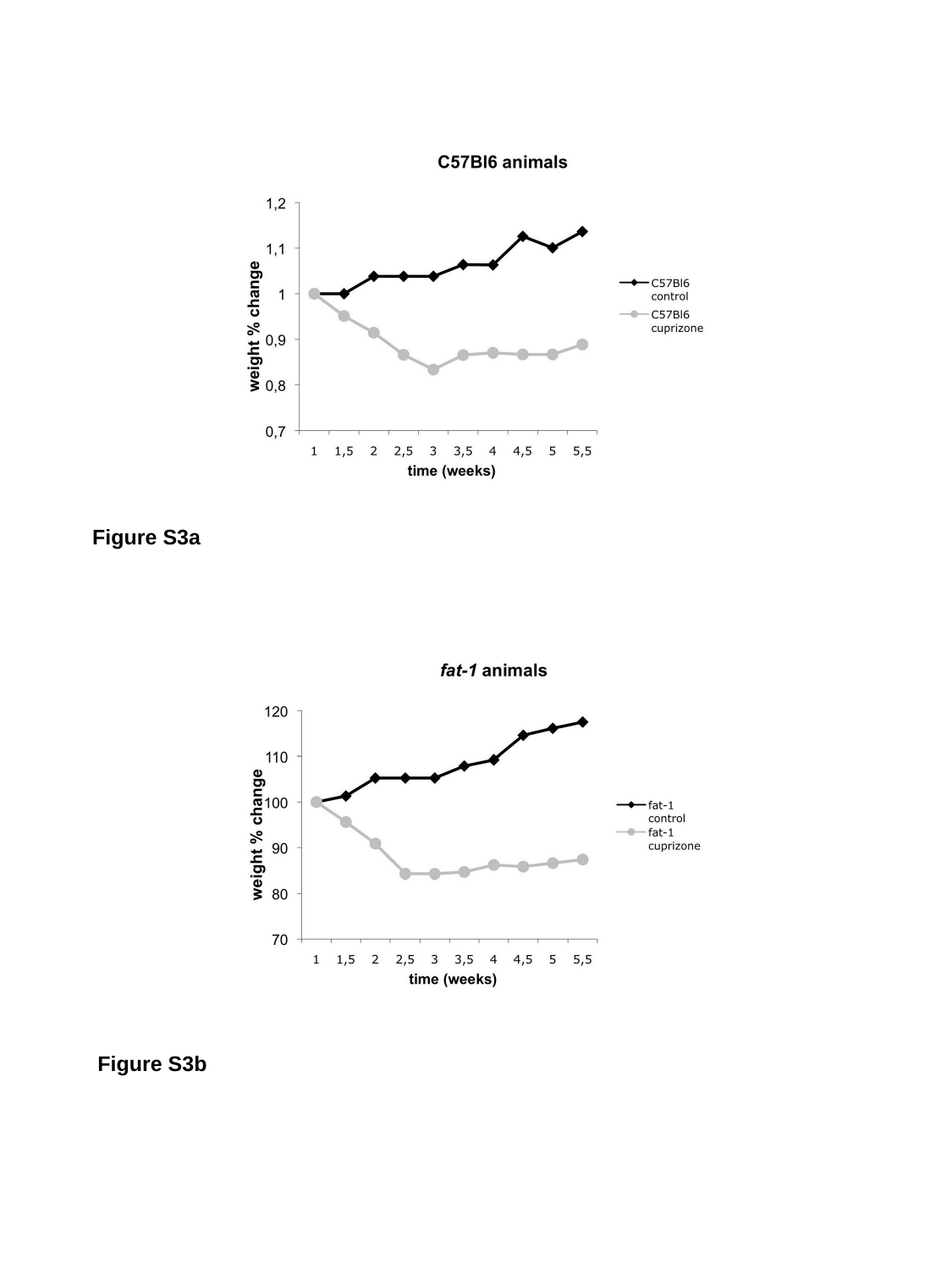

Figure S3a
Figure S3b

Supplement: Supplementary file 3 — Additional file 3: Figure S3. Changes of animal weight in the course of the cuprizone treatment. There are clear differences in the development of animal weight between those animals that had received the cuprizone diet and those that had stayed on a normal one. However, no significant difference could be found between wt and fat-1 animals that belong to the same treatment group. [file 12868_2016_312_MOESM3_ESM.pptx]
